# The influence of depression on risk development of acute cardiovascular diseases in the female population aged 25–64 in Russia

**DOI:** 10.3402/ijch.v72i0.21223

**Published:** 2013-08-05

**Authors:** Valery V. Gafarov, Dmitry O. Panov, Elena A. Gromova, Igor V. Gagulin, Almira V. Gafarova

**Affiliations:** 1 Collaborative Laboratory of Epidemiology Cardiovascular Diseases Siberian Branch of the Russian Academy of Medical Sciences, Novosibirsk, Russia; 2 Laboratory of Psychological and Sociological Issues of Internal Medicine, Institute of Internal Medicine Siberian Branch of the Russian Academy of Medical Sciences, Novosibirsk, Russia

**Keywords:** depression, awareness, attitude to the health, relative risk, myocardial infarction, stroke

## Abstract

**Background:**

Recent studies showed that depression was an independent predictor of mortality from cardio-vascular disease in healthy women.

**Objective:**

To explore the effect of depression (D) on relative risk (RR) of myocardial infarction (MI) and stroke for 16 years (1995–2010) in the female population aged 25–64 years from Novosibirsk, Russia.

**Materials and methods:**

Under the third screening of the WHO “MONICA-psychosocial” (MOPSY) programme, a cohort of women aged 25–64 years (N=560) was surveyed. Women were followed for 16 years for the incidence of MI and stroke (1995–2010). D was measured at the baseline examination by means of test “MOPSY”. Participants having stroke, MI, arterial hypertension, coronary artery diseases and diabetes in their medical history at the baseline were excluded from this analysis.

**Results:**

The prevalence of D in women aged 25–64 years was 55.2%. With the growth of D levels, positive self-rated health reduced and almost 100% of those women have complaints about their health, but considered the care of their health insufficient. Women with major D significantly extended negative behavioral habits: smoking and unsuccessful attempts to give up, low physical activity, and less likely to follow a diet (healthy food). Major D associated with high job strain and family stress. Relative risk (RR) of MI development in women with D during 16 years of study was higher in 2.53 cases (p<0.05) and risk of stroke was higher in 4.63 cases (p<0.05).

**Conclusions:**

The prevalence of D in women aged 25–64 years was >50%. Women with D had a 2.53-fold risk of MI and 4.63-fold risk of stroke during the 16 years of follow-up.

Recent studies showed that depression (D) was an independent predictor of mortality from cardiovascular disease (CVD) in healthy women (1). Gender roles and social traditions reinforced this pathological pattern in patients with adverse affects (2). In terms of social strains, there were high levels of negative psychosocial factors and social deprivation in Russia during the socio-economic crisis; consequently, a high prevalence of negative behavioral habits was observed in the Russian population over 2 decades (3–5). Our previous studies confirmed that psychosocial factors can act as independent factors in CVD development (3). It was necessary to study the effect of D as an independent factor on the risk of acute CVD and to indicate the social gradient, awareness and attitude to health, and prevention in open female population aged 25–64 years.

## Materials and methods

Within the framework of the third screening (1994) of the WHO programme “Multinational Monitoring of Trends and Determinants of Cardiovascular Disease” (MONICA) and subprogramme “MONICA-psychosocial (MOPSY)” (6), a random representative sample of women aged 25–64 years (n=870, Table I) from one of Novosibirsk's districts was carried out. This representative sample was generated on the basis of the electoral lists of citizens using a table of random numbers. The list contained only Russian citizens (who can vote), mostly European Russians, who tend to stay in the region (>90% of the local population), and the dropout rate was relatively small due to the low migration rate (about 5%).

**Table I:** Demographic data surveyed in the third screening of the WHO MONICA, 1994

Age groups	n	%
25–34	214	24.6
35–44	192	22.1
45–54	231	26.6
55–64	233	26.8
25–64	870	100.0

The cohort (N=560, Table II) was formed after excluding women with the following: hypertension (270 cases), stroke (7 cases), myocardial infarction (MI) (2 cases), coronary artery disease without MI (11 cases), diabetes (15 cases), as well as diabetes mellitus (5 cases). The study identified the first cases of emerged MI, stroke as the “end point”. For the 16 years (1995–2010) of study, 35 stroke incidences (Table III) were registered by means of examination, analysis of medical histories, cards and death certificates (5). Fifteen MI incidences (Table III) were determined using WHO programme called “Register of Acuter MI” (7).

**Table II:** Cohort of women with depression but without CVD were followed for 16 years in the third screening of the WHO MONICA, 1994

	Depression							
	
	Major level		Average level		No		All	
				
Age groups	n	%	n	%	n	%	n	%
25–34	20	10.8	82	44.1	84	45.7	186	100
35–44	22	14.3	63	40.9	69	44.8	154	100
45–54	11	10.2	46	42.6	51	47.2	108	100
55–64	14	12.5	51	45.5	47	42.0	112	100
25–64	67	12.0	242	43.2	251	44.8	560	100

**Table III:** The incidence of cardiovascular events (myocardial infarction, stroke) over 16 years for women aged 25–64 years with depression

	Depression		Myocardial infarction		Stroke	
			
	n	%	n	%	n	%
Yes	309	55.2	11	73.3	21	60
No	251	44.8	4	26.7	14	40
All	560	100	15	100	35	100

This survey was performed using the standard methods accepted in the “MONICA study” protocol. The programme of psychosocial screening examinations included the registration of social characteristics, such as marital status, level of degree, professional class and psychosocial tests.

Levels of D were measured using the questionnaire MOPSY (subscale D) (8), which consisted of 15 statements. The answer on each statement had 2 grades: “agree”, “disagree”. Severity of D was assessed as: no depression (ND), average level (AD), major (MD). Questionnaire “Awareness and attitude towards the health” (9) was used for assessment of self-rated health and attitude towards behavioral habits and evaluation of job and family stress.

Questions regarding diet (healthy food), smoking and physical activity were used for the evaluation of the participants’ attitude towards their own health as part of the “MONICA study” protocol.

Statistical processing was fulfilled by means of programme pack SPSS version 11.5. Cox-proportional regression model used estimated relative risk (hazard ratio – HR) taking into account different time intervals: 5 years from the onset of this study, 10 years from the onset of this study and 16 years from the onset of this study. The Chi-square test used to test the statistical significance of differences between groups (χ^2^); p<0.05 was considered statistically significant. D was considered as an independent factor; age standardization was performed in order to eliminate the age effect.

## Results

Levels of D in the female population aged 25–64 years in 1994 were as follows: average D – 43.2%, major D –12%.

The structure of marital status in a cohort of women with D and MI is as follows: married – 66.7%; divorced – 33.3%. In women with D and stroke incidence, the structure of marital status was: never been married – 16.7%; married – 83.3%.

More than half of the participants (66.7%) with D and MI had incomplete higher/vocational education; 33.3% had incomplete high-school education. Those with D and stroke (66.7%) had incomplete higher/vocational education; 16.7% had incomplete high-school education and 16.7% had incomplete elementary education.

Professional status in women with D and MI was as follows: 33.3% were managers, accounted for the remaining share of retirees (55-year-old women). Professional status in women with D and stroke was: 16.7% – heads; 33.3% – hard physical labour; 16.7% – moderate physical labour; and 33.3% – easy physical labour.

There were 6.3% incidents of stroke, 2.7% of MI in women after 16 years of follow-up.

HR of MI incidence in the group with D during 16 years of follow-up was 2.5 times higher (HR=2.53; 95% CI=1.26–24.34; p<0.05) compared to the group without D. The risk of stroke for the same period was more than 4 times higher for participants with D than without D (HR=4.63; 95% CI=1.03–20.89; p<0.05).

An association between D and awareness and attitude towards health showed 7-fold fall in positive health estimation as «healthy» (MD – 4%, ND – 28.2%; χ^2^=41.47; df=8; p<0.001). There was also an increase in negative self-rated health as «sick». Overwhelming, the majority of women with MD had complaints about their health (χ^2^=17.42; df=6; p<0.01), and insufficient care about their health (ND – 58%, MD – 80%; χ^2^=17.69; df=4; p<0.01). Additionally, they rarely believed in the possibility of medicine to successfully treat heart diseases (MD – 28%, ND – 38.6%; χ^2^=23.18; df=8; p<0.01), and were more likely to report a high probability to be ill in the next 5–10 years (MD – 64%, ND – 47.3%; χ^2^=10.51; df=4; p<0.05).

Woman with MD were less likely to continue to work and seek medical help in answering the question “If you feel not so good at workplace, what do you do?” (MD – 39.6 and 29.1%, ND – 59.6 and 8.2%, respectively; χ^2^=15.8; df=4; p<0.01).

In relation to smoking, women with MD showed a trend towards having more than a low frequency of “never smokers” (ND – 75.9%, MD – 68%) and a higher frequency of “quitters” (ND – 7.5%, MD – 14%), “smoking, attempt unsuccessfully to quit” (ND – 2.1%, MD – 6%), than those without D. In relation to diet, it was evident that women with D rarely followed the diet and had more unsuccessful attempts to follow it (MD – 4.1 and 22.5%, ND – 5.5 and 13.8%, respectively; χ^2^=17.87; df=8; p<0.05).

A study of stress in the family indicated that with a growth in D levels, the number of conflicts in the family also increased (MD – 70%, AD – 59.4%, ND – 50.3%; χ^2^=16.46; df=6; p<0.05). In the presence of D, women often said “anything disturb their rest at home” (MD – 56%, AD – 50.3%, ND – 39.4%; χ^2^=6.68; df=3; p<0.05). There was a tendency towards an increase in severe illness rates or deaths of relatives during the last year, as well as changes in marital status in women with MD.

A study of job stress showed the following: women with major D more likely to change their specialty compared to those with AD and ND (64.2, 43.4 and 38.1%, respectively; χ^2^=12.28; df=4; p<0.05); and aimed to reduce the work load and additional work tasks (31.1, 11.7 and 6.1%, respectively; χ^2^=27.26; df=6; p<0.001). Indicator of job responsibility as “high” fell and rate of “low responsibilities” increased with the growth of D levels (MD – 27.3 and 15.9%; ND – 57.6 and 10%, respectively; χ^2^=19.9; df=8; p<0.05). Individuals with D had higher levels of reducing job responsibility in the last year (χ^2^=19.78; df=6; p<0.05). Women with MD were 5 times more likely to show a decline in working capacity during the last year (60.4%; χ^2^=48.9; df=6; p<0.001).

In relation to physical activity, the following trends were observed with an increase in D: the number of participants, who exercised regularly (MD – 4%, ND – 10.8%) decreased; however, the number of women who over the last year had become less active, increased (MD - 34.7%, ND – 14.6%; χ^2^=14.53; df=6; p<0.01) as well as those who understood that they had low physical activity level (MD – 55.1%, ND – 7.5%; χ^2^ =64.36; df=8; p<0.001).

## Discussion

The results showed a high prevalence of D (55.2%) in the female population aged 25–64 years in Russia that was significantly higher than in Europe and the United States (10, 11). In our opinion, such differences were due to well-known events in Russia in 1994, when the population had high levels of stress and psychosocial factors (6, 12, 13). It was associated with the period of changes in social foundations during the transition to another socio-economic structure.

Married women were more likely to have D and develop infarction, stroke, than unmarried and divorced women. D and incidences of MI and stroke were not associated with a higher education, but they were associated with the occupational levels of the participants: low-level executives (managers) and physical workers. This evidence is confirmed by recent studies, where women with higher levels of education have a lower risk of morbidity and mortality from MI and stroke (14–16). With regard to occupational class MI and stroke development in women with D in social class “manager” caused by role conflict “family vs. career”, but in the “manual labour” category, it was due to an imbalance in the model of “demand/control” (17).

Negative health estimation such as “not well”, “sick” increased with the growth of D levels. Associations of D with poor self-rated health in women were obtained in other epidemiological studies (1, 18). Awareness of preventive methods was also lower in women with D (18).

Women with MD had significant levels of job and family stress. The presence of D reduced the flexibility of the individual to “stressors” causing a dysregulation of the central nervous system leading to the formation of the lack of achievement, which results in less pleasure. According to previous publications, family stress and role function independently associated with symptoms of D in female population (19). In addition, an increase in family obligations was associated with a higher risk of coronary heart disease (20, 21).

Our findings showed that women with D expressed more negative behavioral characteristics than women without D. Women with MD often unsuccessfully tried to quit smoking, followed diets less and did physical exercise in comparison with those without D. It was known that the relationship between smoking and D was greater for women than for men (22). Woman with D were more likely to be smokers and pay less attention to physical activity (23).

The presence of D in women aged 25–64 years associated with a 2.5- and 4.6-fold increase of MI and stroke risk incidence after 16 years from screening baseline. Similar data were replicated in the recent studies that showed an independent effect of D on the risk of CVD in women with no history of it (24–26).

## Conclusions

- A high prevalence of D was determined in a random representative sample of women aged 25–64 years from Novosibirsk, Russia.
- The presence of major D in the female population aged 25–64 years reduced self-rated health and awareness of their health, weakened behavioral profile and increased levels of job and family stress.
- It was found that D had increased a relative risk of MI and stroke incidence by about 2.5- and 4.6-fold after 16 years of follow-up period.
- A higher incidence of MI and stroke was found in women aged 25–64 years with D in the following categories: “married”, “high-school education”, “head” and “manual labour”.
